# Grounded theory-based model of the influence of digital communication on handicraft intangible cultural heritage

**DOI:** 10.1186/s40494-022-00760-z

**Published:** 2022-08-08

**Authors:** Jia Li

**Affiliations:** 1 School of Journalism and Communication, Sichuan International Studies University, Chongqing, 400031 China; 2 School of Design, Silla University, Busan, 46958 Korea

**Keywords:** Digital communication, Handicraft ICH, Influencing factors, Theoretical model, Grounded theory

## Abstract

Digital technologies have altered social interaction and information transmission, creating new cultural communication media and models. Despite extensive investigations into the effect of digital communication on handicraft intangible cultural heritage (ICH), research on the relationship between the factors that influence the digital communication of handicraft ICH is relatively scarce. This study used open-ended interviews and textual analysis to obtain data concerning these influencing factors. A grounded theory approach found that five core factors have significant effects on the digital communication of handicraft ICH. These are digital communication awareness, cultural communication initiative, adaptability of digital technology, audience acceptance and cognition, and uncontrollable factors. Furthermore, a theoretical “awareness-behavior-attitude” model based on an analysis of the relationships between the five factors was constructed. Finally, the action mechanism of the theoretical model was identified and tested, revealing that the action modes of each influencing factor were inconsistent. Antecedent factors were directly proportional to awareness-behavior, situational factors were inversely proportional to awareness-behavior, and regulatory factors were inversely proportional to behavior-attitude. In addition, situational factors and regulatory factors were found to have a positive impact on antecedent factors.

**Supplementary Information:**

The online version contains supplementary material available at 10.1186/s40494-022-00760-z.

## Introduction

Intangible cultural heritage (ICH) consists of traditional and ongoing cultural representations and practices, including oral traditions, social practices, rituals, festive events, and knowledge of the environment, and the knowledge, skills and social practices required to produce art and handicrafts [1]. The deep penetration of new technologies and new media has made the dissemination of ICH through digital means increasingly widely practiced and accepted. Projects such as American Memory, Memory of the World [2], and the Digital Protection Project of Chinese Intangible Cultural Heritage [3] play an important role in promoting cultural identity and preserving traditional practices. The use of these technologies also has the potential to impart economic and cultural benefits on ICH practitioners [4–6] which will ultimately contribute to the survival of these practices. One important aspect of ICH has been the acceleration of the digital dissemination of traditional handicrafts. Digital technologies such as the Internet, cloud computing, 5G, artificial intelligence, augmented reality, and virtual reality have imbued traditional handicrafts with a new vitality. Handicraft practitioners increasingly use social platforms such as YouTube, Twitter, Facebook, Sina Microblog, and TikTok. For instance, Li Ziqi, named ICH person of the year in China in 2021, is the most highly subscribed YouTube vlogger in China [7]. Moreover, online museums, live broadcast rooms, virtual communities, and other new media are boosting the protection and promotion of traditional handicraft techniques [8–10]. When used to their best advantage, these various platforms and media allow ICH practitioners to share work, participate in craftsmanship communities, and develop effective archives [11], to disseminate skills and practices to new and young audiences [12], and to open up new markets to generate revenue [13]. Therefore, the use of information technology in many aspects of the dissemination and protection of handicraft ICH is proving to be a feasible strategy that aligns with current development trends in networked cultural communication.

However, the digital communication and dissemination of handicraft ICH presents a complex range of problems related to information transmission, audience psychology, and technology. The mode of communication is affected by a number of variables, including the drivers of digital communication, the origin of the factors influencing digital communication, and the interactive relationship between those factors. Research in Portugal [14] found that one of the biggest challenges is the adoption of digital media by handicraft practitioners. Even when digital media are adopted, there remains the challenge of effectively communicating intangible and person-specific knowledge to distant audiences [15]. The digital communication of handicraft ICH is comprised of syntheses and complexities. While communication practices can be enriching, can expand practitioner communities and cultural dissemination, and can meet personalized audience needs, it also presents a number of practical challenges. Hou et al. [15] argue that most digital interventions into handicraft ICH pay greater attention to the preservation of material-based objects than to reviving and representing the living nature of ICH. This makes it difficult for audiences to identify with the more intangible content of digital scenarios [16–18].

This paper seeks to address some of the shortcomings in our understanding of the use of digital technologies in the practice and dissemination of handicraft ICH. It investigates the influencing factors and action mechanisms at play in the digital communication of handicraft ICH. Understanding these factors and mechanisms has practical significance for the formulation of strategies that will promote the communication and successful dissemination of handicraft ICH. While much existing literature focuses on independent influencing factors in the digital environment, there has been far less research exploring more complex digital and communication relationships [15, 19]. This research, uses grounded theory to develop a model of the digital communication of handicraft ICH from the perspective of influencing factors and action mechanisms. The grounded theory approach allows us to understand the world of digital communication of handicraft ICH from the perspective of practitioners and consumers and to explore the complex relationships between the different actors and factors in that world. Expanding our understanding of digital communication and dissemination of ICH through the development of a relational model opens up new avenues for research.

## Existing research and shortcomings: two perspectives

The digital communication of handicraft ICH is a relatively new, and still small, interdisciplinary field, presenting space for a great deal of further academic investigation in an area of rapidly developing technology [20]. Researchers have, for the most part, focused on the development of handicraft ICH digital content and the effects of information technology on the protection of traditional handicrafts [11, 21]. Few studies, however, specifically focus on the environment in which handicraft ICH is digitally disseminated. The digital communication of handicraft ICH research generally adopts one of two approaches: (1) an analysis of media innovation in handicraft ICH digital communication or (2) an analysis of the cognitive effects of digital information on audiences from the perspective of behavioral psychology.

The first approach explores how digital media overcome spatiotemporal limits to share and disseminate content, and the use of artificial intelligence technology to deep mine and optimize the use of cultural resource data [22, 23]. The use of such data facilitates wider communication and leads to personalized handicraft ICH display effects, leading to enriched traditional handicraft display techniques and communication forms [22]. Digital platforms, for example, allow for the creation of multi-dimensional contacts and for the processing of experience through different forms of interaction. Niche customization platforms, such as Etsy, and professional integrated platforms, such as JD ICH, support the publishing and selling of handicrafts [24]. Yu and Wang [23] have explored the use of 5G technology to reconstruct communication scenarios incorporating subject, content, information form, the ecological environment, and user experience. Using 5G technology, virtual reality, augmented reality, and reality can be organically integrated to realize experiential communication. With the maturity of VR and other virtual reality technologies, for example, artificial intelligence technology allows for speech recognition, image scanning, and intelligent modeling to build handicraft ICH simulation scenes which can then be displayed and disseminated via social media [25].

Jin, meanwhile, suggests using digital media to better protect handicraft ICH and proposes the visualization communication of handicraft ICH through digital movies, animation, documentaries, and applications, all of which will enhance the digital media channels through which the public can access and understand handicraft ICH [26]. Research is also ongoing on the construction of a collaborative innovation model of handicraft ICH based on cloud technology with the aim of developing cooperation mechanisms, technical support, and the theoretical construction of a digital cloud platform to promote the networked dissemination of handicraft ICH [25]. For instance, researchers have experimented with creating shared digital platforms for handicraft practitioners to promote and sell their products [14] or to create online communities of handicraft practitioners [11]. Many digital communication methods based on handicraft ICH have been successfully implemented by taking advantage of new digital technology, mobile Internet terminals, and intelligent devices. Digital communication methods take three forms: (1) 3D simulation, panoramic digital animation, short video communities, and online virtual museums such as, for example, the British Museum’s 3D animation on Facebook of the Chinese Ming Dynasty landscape work *Autumn Forest Reading*, which overcomes language and text communication barriers, and establishes an easily understood communication style [27, 28]; (2) Interactive experiences using mobile tools such as smart devices, such as, for example, TopSmart, a service platform for smart glasses technology solutions that has teamed up with Suzhou Embroidery (a handicraft ICH). Using TopSmart AR technology, viewers can experience dynamically displayed traditional embroidery craftsmanship [29]; (3) Immersive game platforms, theme applications, and HTML5 interactive mini-games spread on social media such as, for example, *Tianya Mingyue Knife* which integrates Suzhou Taohuawu New Year wood-block prints, a form of handicraft ICH, into the game to build scenery and story content. Players can choose a unique traditional cultural perspective and achieve living communication in the native cultural environment [30, 31].

The digital communication of handicraft ICH is dependent on a series of variables, and media innovation is only one of these. However, most existing studies focus on the promotion of digital technology only while ignoring other variables.

The second approach explores how the use of digital media influences audience cognition of handicraft ICH content. Attitude theory from the field of psychology and the technology acceptance model have been used to analyze the influence of external digital environmental factors on internal factors such as user beliefs, attitudes, and intentions [32, 33]. Related findings suggest that audiences are relatively passive in their reception of digital content display, such as traditional techniques or cultural scenes, and that there is a causal relationship between audience attitudes toward the use of digital ICH platforms and actual use behavior [22]. Meanwhile, ethnographic research has been conducted on the status of audience acceptance of handicraft ICH digital information, while quantitative and cognitive effect analyses have been conducted on user samples of new media platforms such as handicraft ICH promotional websites, official accounts, and blogs. This research has found that attracting the attention of users, and especially of young people, through games and animation guarantees the sustainable dissemination of handicraft ICH [34]. The combination of digital experience and physical activities is the most effective way to enhance audience awareness of handicraft ICH. Developing more practical and interesting auxiliary functions of new media platforms and extending information dissemination to, among other areas, public services, social education, and interactive feedback can greatly improve the depth and breadth of audience cognition of handicraft ICH [35, 36]. Using the Elaboration Likelihood Model and Affect Transfer Theory, Xue and Li [37] have used the handicraft ICH of Suzhou embroidery as the research object to explore the influence of digital information on audience identification and microblog information. Information richness, source credibility, and site popularity have a significant positive impact on audience perceptions and recognition of handicraft ICH [37]. Meanwhile, Venkatesh et al. [38] used questionnaire surveys and offline interviews to understand the cognitive effect of the digital transmission of handicraft ICH on audiences, based on the Unified Theory of Acceptance and Use of Technology model, while Xu and Sun [39] have used structural equation model analysis to establish that outcome expectations, ease of use expectations, the intervention of others, and promotion conditions have a significant impact on public willingness to engage with and make use of handicraft ICH digital information, although moderating variables such as gender, age, and educational background have a certain impact.

From the perspective of behavioral psychology, most existing studies are based on quantitative research and are predominantly from the perspective of audience cognition. Therefore, due to a lack of attention paid to the disseminators of handicraft ICH, digital communication mechanisms are not fully understood.

Most existing studies, therefore, focus on either media innovation or behavioral psychology. They typically focus on the effect of a certain independent variable in the digital environment on the transmission of handicraft ICH, and pay less attention to the digital transmission process and the interaction relationship between each variable. In addition, disseminating the living and personal elements of ICH [15] and creating handicraft interactions that are consistent with user logic [19] remain challenges in the development of techniques to successfully disseminate handicraft ICH.

The present study, therefore, focuses on the variables that influence the digital communication of handicraft ICH. I explored how these variables impact on ongoing engagement with handicraft ICH. Using interview and text analysis data, I used a grounded theory approach to first identify the influencing factors in the digital communication of handicraft ICH and then analyze the relationships between the identified variables. Finally, I constructed a theoretical model of these influencing factors and explained and verified the action mechanism of each factor in the model.

## Data collection: open-ended interviews and text analysis

Grounded theory is a method largely used in the qualitative social sciences. It involves the deep analysis of data that has been systematically collected in order to build a theory grounded in those data. It is often used to develop an understanding of social relationships and processes [40]. I adopted a grounded theory approach to theoretically describe the essence and significance of the phenomenon of the digital dissemination of handicraft ICH and to establish a suitable theory to describe the actual situation. A grounded theory analysis of open-ended interview transcripts and texts helped us to establish the key influencing factors in the digital dissemination of handicraft ICH.

First, to ensure that the study was scientific and the material comprehensive, I used the theoretical sampling method to select specific interviewees according to the development of the theory. Theoretical sampling is the key procedure in grounded theory, used to integrate data collection, coding, and theory construction into a continuous closed-loop process. Sampling ends when new data no longer produce new theories [41, 42]. Given that the interview content covered topics such as “communication subjects, digital media, cultural conflicts, technical barriers, and the content-technology relationship,” respondents were required to have an understanding of the digital communication of handicraft ICH. Therefore, starting from the three inclusion criteria of professional background, work experience, and hobbies, the target interviewees were placed into three interview groups, namely, scholars, practitioners, and enthusiasts; in total, nine people (three in each group) were interviewed face-to-face or online. Each interview lasted approximately half an hour. All interviews were conducted in Chinese. Table 1 shows the basic information of the interviewees.

**Table 1:** Interviewee information

S/N	Group	Occupation	Age	Education	Characteristics
F01	Scholars	Associate professor	51	PhD	He has visited many folk museums in China and South Korea, and is interested in cultural conflicts in handicraft ICH communication
F02		University lecturer	40	PhD	She is engaged in a variety of handicraft ICH, including embroidery and paper cutting, and is fascinated by handicraft videos, such as those of Li Ziqi
F03		Professor	44	Master’s	She has participated in many cultural and craft protection projects, and teaches university courses related to the digital communication of traditional handicrafts
F04	Practitioners	Handicraft operator	46	Master’s	He runs an art company that aims to disseminate ICH
F05		Marketing Specialist	42	Bachelor’s	He is an enthusiast of the use of new technology in handicraft production and is experienced in digital exhibition and online interaction
F06		Self-media operator	32	Bachelor’s	He has worked for the Chinese online platforms Tencent and iqiyi, and has made ICH videos and documentaries. In particular, he has participated in creating documentaries on the ICH of Southern Fujian
F07	Enthusiasts	International student	23	Master’s	He enjoys making handicraft videos to share with friends, and he is a strong enthusiast of AI technology
F08		Unemployed	24	Bachelor’s	She has experienced many traditional skills, including the Guizhou tie dyeing process. She wishes to combine online communication with offline experience
F09		High school teacher (retired)	65	Bachelor’s	She is concerned about the impact of traditional handicrafts on art education and is interested in digital museums

Interviewees were asked a series of open-ended questions on a range of topics related to the digital communication of handicraft ICH. From the production perspective, they were asked to discuss the awareness, abilities, and use of digital media by handicraft practitioners and the technical challenges faced in their use. From the audience perspective, they were asked to consider whether the style of digital communication affects impressions of handicraft ICH, and in what ways it deepens understanding and appreciation of traditional handicrafts. Finally, interviewees were asked to discuss ways to more effectively exploit digital opportunities to the advantage of handicraft ICH.

After interviewing the first group, I analyzed and compared the collected data, and flexibly added new questions to form the outline for the interviews with the second group. After the third group was interviewed, no new problems were found, and the interviews were concluded. Additional file 1: Appendix A (in supplemental material) provides an initial interview outline.

Second, to ensure the data were comprehensive, I conducted a keyword search on the China Knowledge Network (CNKI), a database of Chinese academic and government publications. I used the keywords “handicraft intangible cultural heritage & digital communication” and “traditional handicraft & digital communication” for the time period 2016–2021 as, prior to 2016, there are few relevant papers on this topic and their reference value is limited. I obtained 16 papers from the China Social Science Citation Index (CSSCI) (numbered from P01–P16 in chronological order of publication; see Additional file 1: Appendix B in supplemental material) and then analyzed and organized the content of these papers according to the questions in the interview schedule. When new information was discovered, I returned to the original research and explored it through an iterative process.

Finally, having completed the semi-structured open-ended interviews and textual analysis, I used stratified sampling to select six interview records, and used equidistant sampling for coding analysis and model construction to select 10 materials for textual analysis. The remaining interview records and textual analysis data were retained for model testing.

## Model construction based on grounded theory

Because the digital communication of handicraft ICH is a relatively new field of scholarship, it lacks mature theoretical assumptions and variable research categories. Therefore, I used grounded theory to identify related influencing factors and to construct an action mechanism model to provide both theoretical and practical guidance for future research. Grounded theory, as proposed by Glaser and Strauss [43], is a qualitative method that establishes a theory grounded in the analysis of the data that have been collected [44–47]. It emphasizes the search for core concepts within the collected data, summarizing those concepts and using coding to construct a theoretical framework. Coding is divided into three stages, namely, open coding, axial coding, and selective coding [48, 49].

### Open coding

**Fig. 1:**
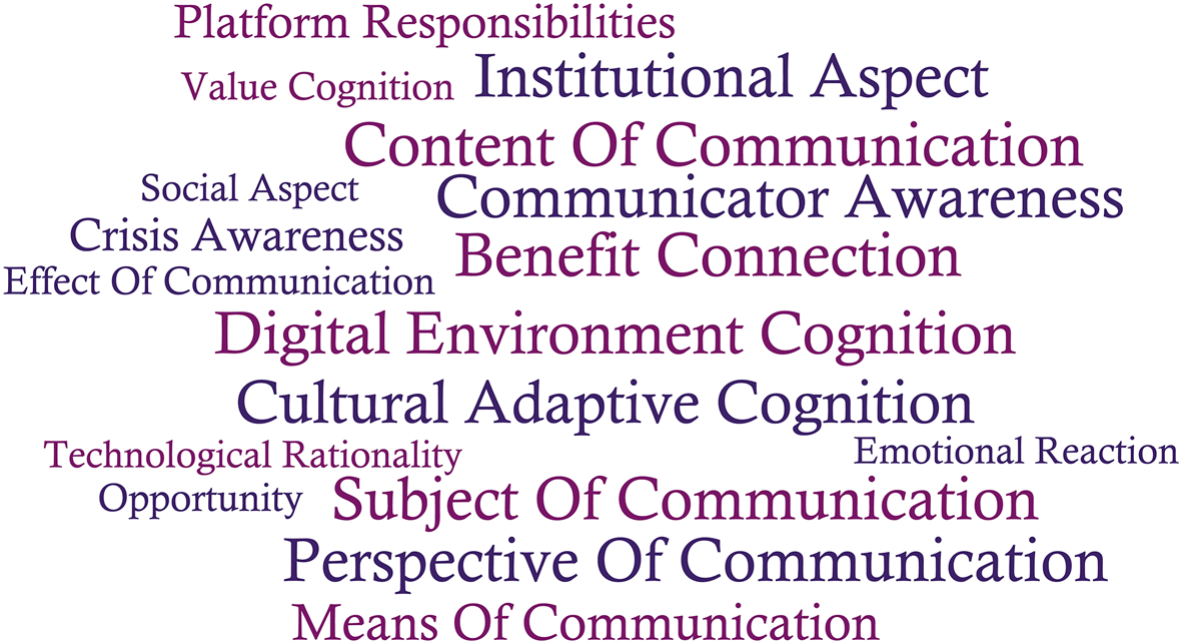
Open coding: 17 initial concept categories

Open coding is a process of sentence-by-sentence coding of the original data. To reduce error and human bias, I mined the respondents’ original statements (with partial fine-tuning and correction) and the textual content for initial concepts. Then, I open coded the six sample interview records and ten sample textual materials. The initial concepts and related representations were very complex, so I categorized these according to overlapping and subordinate relationships, eliminating statements that appeared fewer than three times. Finally, through multiple rounds of open coding analyses, I obtained 17 frequently occurring initial concept categories which are presented in Fig. 1 in the form of a word cloud. Additional file 1: Appendix B contains a list of the open coding categories and original records.

### Axial coding

**Fig. 2:**
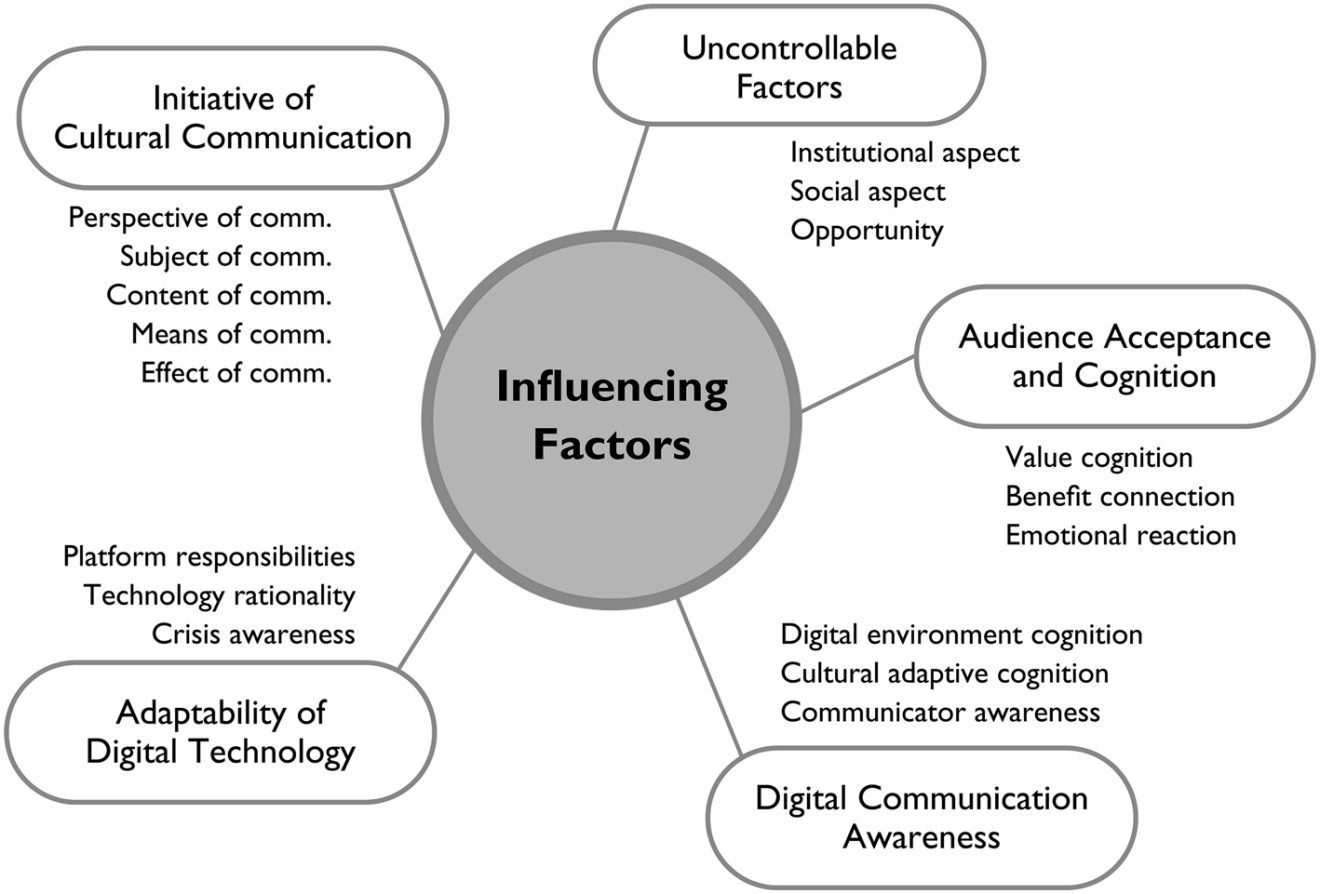
Axial coding: five key concept categories

I next performed axial coding, which involved a clustering analysis of the open coding data. In this way, I merged similar codes to form more general macro concepts. Based on the 17 frequently occurring categories, I analyzed causal and logical relationships between the 17 frequently occurring categories at the conceptual level, and obtained five core concepts: digital communication awareness, cultural communication initiative, adaptability of digital technology, audience acceptance and cognition, and uncontrollable factors (Fig. 2).

During axial coding I also described the phenomenon and the conditions of the digital communication of handicraft ICH by following the relationships and analyzing the potential behavioral context of the five concepts. I then studied the logical and causal relationships between these concepts and finally developed the theoretical structure shown in Table 2. Awareness of the digital communication of handicraft ICH appears to be the internal driving factor directly affecting digital communication behavior and attitudes towards handicraft ICH. The adaptability of digital technology is an internal situational factor, while cultural communication initiative and audience acceptance and cognition are external situational factors affecting the relationship between awareness and behavior; while uncontrollable factors affect the relationship between behavior and attitudes.

**Table 2:** Relational structure based on concepts from axial coding

Concepts	Implication	Relationship
Digital communication awareness	Internal factor and antecedent factor	Awareness–behavior–attitude
Initiative of cultural communication	External situational factor	Awareness–behavior
Adaptability of digital technology	Internal situational factor	Awareness–behavior
Audience acceptance and cognition	External situational factor	Awareness–behavior
Uncontrollable factors	Regulatory factor	Behavior–attitude

### Selective coding

**Fig. 3:**
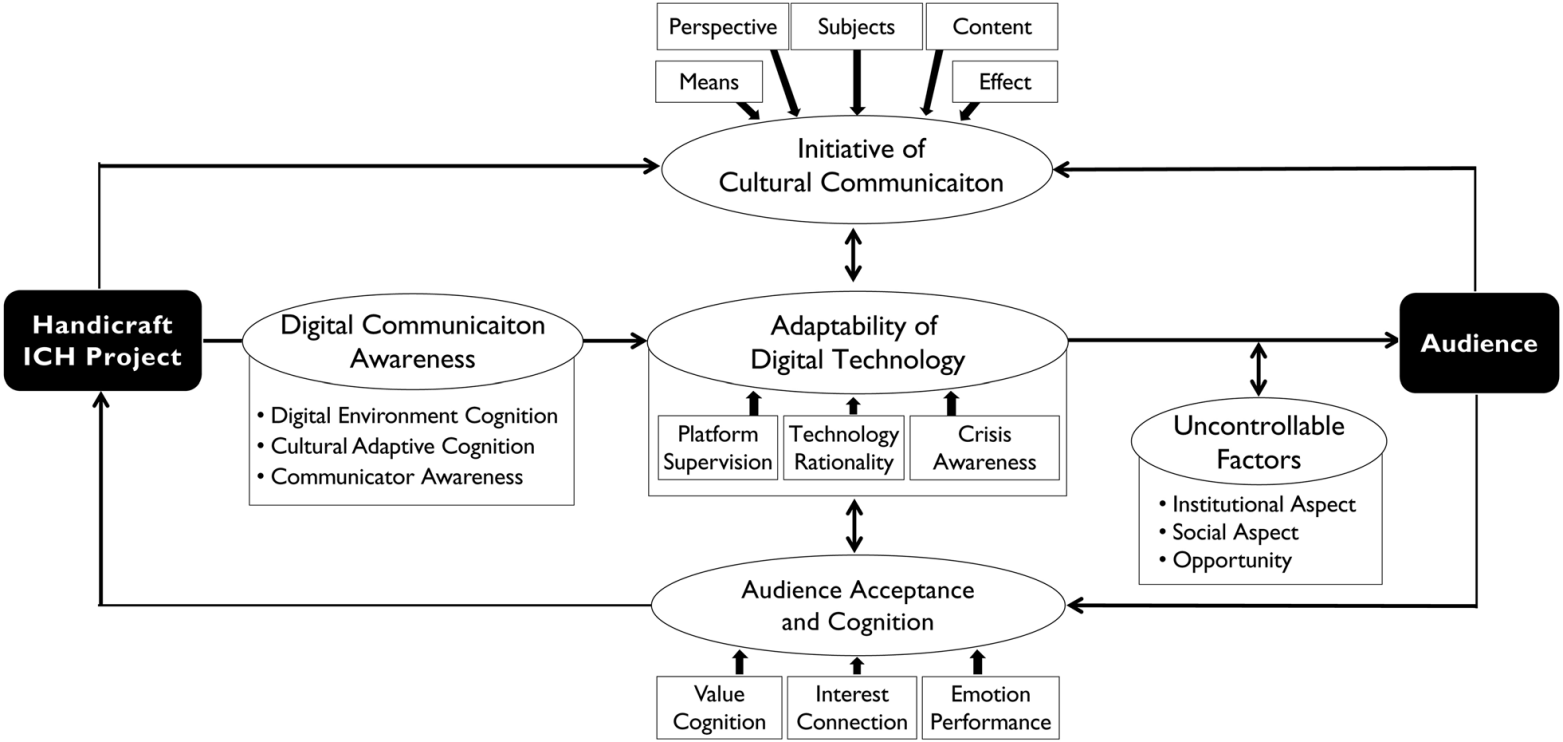
Theoretical “awareness–behavior–attitude” model

Based on the five core concepts and the interaction relationships obtained from the first two levels of coding, I used selective coding to construct a theoretical model of these influencing factors and the mechanisms of digital dissemination of handicraft ICH. I call this the “awareness–behavior–attitude” model (Fig. 3). Awareness refers to a practitioner’s understanding of the digital environment and their ability to use digital technology and digital media to disseminate handicraft ICH. Behavior refers to how a practitioner uses digital technology and media such as, for example, to cooperate with other handicraft ICH practitioners or to work with emerging technologies to create new handicraft ICH experiences. Attitude refers to how practitioners who use digital media participate and interact with others, such as through receiving and responding to feedback to improve the digital communication experience to the benefit of handicraft ICH.

**Fig. 4:**
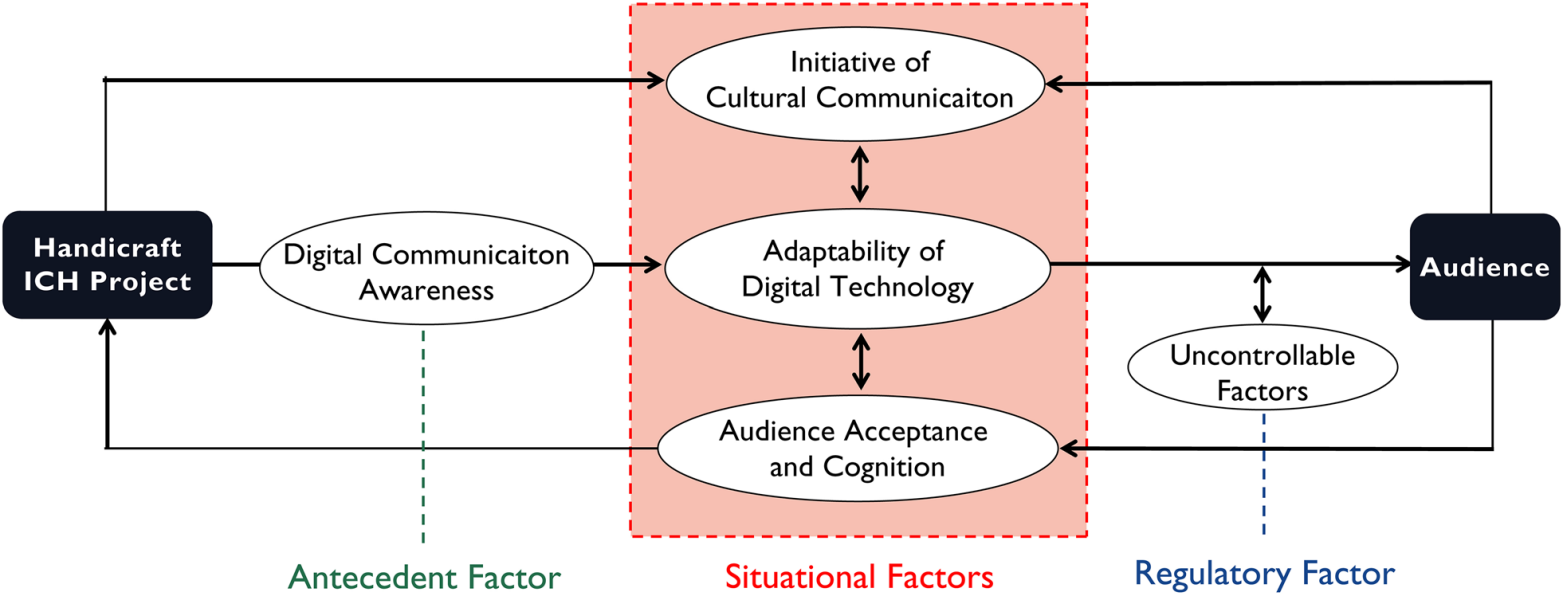
Relationships in the “awareness–behavior–attitude” model

Multiple factors affect the digital communication of handicraft ICH, and each of these factors affect and interact with the others. This model of influencing factors consists of the following relationships (Fig. 4).

The interviewees all highlighted the need to gradually broaden cognitive awareness of the digital environment, of having the cultural foresight to keep pace with the times, and of being sensitive to new technologies and new media. Therefore, digital communication awareness is the internal cause and precondition of the entire communication process. It directly affects the adoption of digital technology and the choice of digital platforms, which in turn affect digital communication activities related to handicraft ICH.

Cultural communication initiatives, audience acceptance and cognition, and the adaptability of digital technology are the internal and external bases for the digital communication of handicraft ICH. The adaptability of digital technology is at the center of the entire digital communication process. It is also an internal basis that relies on digital technology and digital media to connect each element of the awareness–behavior–attitude model. Cultural communication initiative, audience acceptance, and cognition together form the external basis for the digital communication of handcraft ICH. Cultural communication initiative is directly influenced by the communication subjects of handicraft ICH, including both inheritors and experts and virtual subjects such as AI robots and virtual communicators; it also adjusts the direction and intensity of communication through audience feedback. Audience acceptance and cognition is the factor that drives ICH practitioners to adapt their digital communication strategies based on audience feedback which, in turn, directly affects the judgment and behavior of both practitioners and virtual communication subjects.

A number of uncontrollable factors, such as regulating variables, conflicts, and fashion trends, impact the digital communication and dissemination of handicraft ICH. Regulating variables affect the process and effect of digital handicraft ICH communication. One such example is the International Master Craftsmanship plan which the Chinese government introduced in 2016 to support Chinese ICH practitioners at home and abroad. Cultural conflicts, such as over cultural property rights or over regional and national handicraft styles, place pressure and obstacles on the dissemination of ICH. Meanwhile, fashion trends can emphasize certain forms of handicraft ICH over others. For example, Hermes, the French luxury goods manufacturer and retailer, has opened an exclusive store in China that specializes in the sale of ICH products. These uncontrollable factors all lead to unpredictable variables impacting on pace and impact of handicraft ICH communication activities.

### Theoretical saturation test

To test the saturation of the awareness–behavior–attitude model, open coding, axial coding, and selective coding were repeated using data from the remaining three interview transcripts and six texts. The results showed that no new logic or causality was generated in the related categories, and conceptual categories that occurred more than three times were all included in Fig. 1. We inferred, therefore, that the categories in the conceptual model were relatively complete and that the awareness–behavior–attitude model was theoretically saturated.

## Discussion

The awareness–behavior–attitude model can be used to explain the influencing factors and modes of action of the digital communication of handicraft ICH. However, the five influencing factors, influence the modes and action paths of digital handicraft ICH communication in different ways.

### Antecedent factors

Digital communication awareness is the prerequisite for the digital communication of handicraft ICH and is also the internal cause in the entire communication process. Combining the awareness–behavior–attitude model with the findings of existing research reveals the key driving roles of digital environment cognition and cultural adaptability cognition in the digital communication of handicraft ICH. Digital environment cognition results suggest that there are normative principles with regard to the digitization of handicraft ICH in terms of information resources; namely, forming a “digital community” for handicraft ICH information dissemination and building a content activation strategy system. For example, in 2019, Hubei Provincial Museum, the first 5G smart museum in China, disseminated ICH by means of high-definition 4 K live broadcast, VR roaming and 5G holographic projection [50]; Nanjing Yunjin actively promotes the cross-border integration of handicraft ICH culture, digital resources, and other industries through game development, cultural tourism integration, derivatives development, and ICH intellectual property protection [50]; and many museums and art galleries are also actively cooperating with platforms such as Taobao live broadcast, Weibo live broadcast, and WeChat to create "palm museums" and virtual cloud exhibitions [51]. Cultural adaptability cognition results focused on exploring the contemporary value of traditional handicraft culture and its attendant historical memory, cultural characteristics, practical values, and aesthetic significance, and on building an open and innovative digital handicraft ICH creative cultural industry. For example, the creation of the Digital Dunhuang has resulted in the preservation and sustainable use of more than 30% of Dunhuang’s Mogao grottos. The repository was launched in Chinese in 2016 and in English in 2017, and has been visited more than 10 million times. Meanwhile, elements of Dunhuang culture have been digitally transformed, and a series of cultural creations have been developed [24]. However, it should be noted that digitalization may lead to the disappearance of randomness, and naturally occurring irregularity and subtle changes of the process need to be preserved, to better support the inheritance of the cultural value of handicrafts [52].

However, overall, relatively few studies explore handicraft practitioner awareness. Based on our interviews and textual analysis, we found that awareness greatly affects the structure of the digital communication of handicraft ICH, which in turn affects the consistency of awareness–behavior. Although the consistency of awareness–behavior is directly proportional to the level of digital communication awareness, a simple linear relationship does not exist between the two. Only when the disseminators of handicraft ICH are deeply aware of the value of digital communication can they ensure the smooth development of the communication process. Based on the model, we predict a significant increase in digital communication awareness among handicraft ICH practitioners who are more rational about their craft and who display more initiative. This may also explain why many handicraft ICH projects continue to cause cultural conflict due to improper practice when attempting to engage in digital communication. As one interviewee pointed out, “These craftsmen and communicators are still thinking about these issues in a traditional way, so why can’t they change the way of communication?” (F03), while another said “I feel that technicians do technical work while cultural scholars only talk about theories, and audiences feel bored after watching the video without any sense of empathy” (F07). In other words, technicians do not care about content, scholars are divorced from practice, and digital content needs to be made attractive to young audiences.

### Situational factors

Of the situational factors, the adaptability of digital technology is the internal situational factor. It is the media factor of the behavior stage in the "awareness–behavior–attitude" model and, therefore, the core of the entire digital communication process. These factors include internal situational conditions determined by platform responsibilities, technical rationality, and crisis awareness. Initiative of cultural communication is an external situational factor, and is the disseminator factor of the behavior stage in the awareness–behavior–attitude model, so it is at the core of competitiveness. It includes communication perspective, communication subject, communication content, means of communication, and effect of communication. It is directly affected by the subject of handicraft ICH communication, as well as by audience perceptions and attitudes. Audience acceptance and cognition is an external situational factor, and the consumption factor of the behavior stage of the model. It is determined by whether audiences benefit from digital content and their emotional reaction to it. It includes value cognition, interest connection, and emotion performance. It is also affected by audience psychology and behavior [19] while it directly affects the mode of communication that handicraft ICH practitioners choose.

Situational variables moderate the direction and strength of awareness–behavior. Previous research on the initiative of cultural communication focused on intelligent creation and cultural sharing from the perspective of digital information management and dissemination [21]. In light of this, we must consider the management, platform, and strategy for digitally communicating handicraft ICH, as well as the multimedia and interactive development of digital handicraft ICH products. Research on the adaptability of digital technology has predominantly been from a computer science perspective, emphasizing the cultural innovation required to establish an intelligent digital ecology [53]. Such research reveals three trends: the open sharing of traditional handicraft digital resources, the intelligent upgrading of methods and tools in the process of handicraft creativity, and the multimodal integration of handicraft cultural experiences. Meanwhile, audience acceptance and cognition research has predominantly been from a psychological perspective, emphasizing the driving factors and dynamic effects of audience acceptance of digital forms of handicraft ICH [54]. This form of research has used digital virtual characters, virtual scenes, and AR to conduct user evaluations to test virtual experiences of and learning about traditional handicrafts. However, no studies have comprehensively considered cultural communication initiative, the adaptability of digital technology, or audience acceptance and cognition.

From this study, we infer that when the influence of three situational variables is neutral or weak, handicraft ICH digital communication activities are primarily affected by digital communication awareness, and the connection between awareness and behavior is strongest. When the influence of three situational variables is significant (i.e., extremely favorable or unfavorable), such as where there is a rather high cultural communication initiative or when there is unsatisfactory audience acceptance and cognition, it may greatly affect (i.e., promote or inhibit) the digital communication of handicraft ICH; the connection between awareness and behavior is significantly weakened and the influence of internal and external situational factors on digital communication activities increases significantly.

Figure 4 shows that situational factors (both internal and external) belong to the behavior stage and digital communication awareness belongs to the awareness stage. Therefore, we believed that situational factors are directly affected by the orientation and intensity of digital communication awareness. When digital communication awareness is weak (e.g., when the practitioner has limited digital environment cognition or cultural adaptive cognition), the moderating effect of situational factors becomes significant. However, when the communicators of handicraft ICH have strong digital communication awareness, the moderating effect of these internal and external situational factors is weakened. One interviewee stated, “After watching videos and animations of handicraft intangible cultural heritage, I just liked them, but rarely forwarded them or commented” (F06), suggesting that the situational factors of the initiative of cultural communication and audience acceptance and cognition are weak. Meanwhile, another interviewee responded, “The digital transformation of traditional culture requires a process. We should be tolerant of the conflict between the reality of traditional handicrafts and the virtuality of digital experience” (F02).

### Regulatory factors

Uncontrollable factors moderate the direction and intensity of behavior–attitude by affecting the relationship between digital communication behavior and the generation of audience attitudes. A number of previous studies have explored regulatory factors. Li [54] analyzed the Chinese Cultural Digitization and International Communication Project, and proposed that lessons from that experience could be applied to disseminating Chinese Naxi-Dongba culture; i.e., the connection and transformation of real space and virtual space should meet under one overall cultural output strategy [44]. Meanwhile, Xu and Sun [39] found that, among the factors affecting public will and behavior in digital communication of handicraft ICH, moderating variables, such as gender, age, and education, had positive correlations [28]. Chen and Ma [5] considered opportunities through an exploration of digital content packaging and live new-media broadcasting for craftsmen in the context of COVID-19 [2]. These studies highlight the need to reasonably examine the digital environment, respond in a timely manner, and develop corresponding communication strategies.

When the influence of regulatory factors is significant, audience attitudes toward the digital dissemination of handicraft ICH is strengthened or weakened. For example, on the basis of a public welfare project jointly launched by Starbucks and the China Women’s Development Foundation, Starbucks and Guizhou Danzhai Batik Technology jointly launched China’s first intangible cultural heritage experience store in 2021, integrating the cultural experience for customers in unexpected ways. The connection of behavior–attitude is then weakened, and the influence of regulatory factors on digital communication attitude increases significantly. An increase in uncontrollable factors also prompts the disseminators of handicraft ICH to psychologically re-examine their attitudes toward the digital environment. As one interviewee stated, “Just like Li Ziqi’s live broadcast of production videos of various crafts, sometimes there is a lot of controversy. Today’s digital content production methods and new media choices cannot please everyone. But in any case, the premise [of online communication] is that it correctly conveys ideas” (F01), while another stated, “In junior high school, we went to the craft base to practice every week. Once there was any new digital experience project, we would be very happy and hoped to go again next time” (F08).

## Conclusions

Based on interviews and textual analysis, this study used grounded theory to construct a theoretical model of influencing factors and mechanisms for the digital dissemination of handicraft ICH. We identified five core concepts that have a significant impact on the digital dissemination of handicraft ICH. These are digital communication awareness, cultural communication initiative, adaptability of digital technology, audience acceptance and cognition, and uncontrollable factors. We then analyzed the structural relationships between these factors and proposed a mechanism model of awareness-behavior-attitude. We conclude that digital communication awareness is the antecedent factor for the digital communication of handicraft ICH, for the adaptability of digital technology, and for cultural communication initiative; audience acceptance and cognition are internal and external situational variables in the awareness–behavior relationship, and uncontrollable factors moderate behavior–attitude.

The theoretical model of influencing factors and the mechanism for the digital communication of handicraft ICH (i.e., the awareness–behavior–attitude model) that we have proposed is significant in the theoretical construction of the digital dissemination of handicraft ICH. This research used grounded theory to develop a model of the digital communication of handicraft ICH from the perspective of influencing factors and action mechanisms. The grounded theory approach allows for an understanding of the world of digital communication of handicraft ICH from the perspective of practitioners and consumers and an exploration of the complex relationships between the different actors and factors in that world. Expanding our understanding of digital ICH communication and dissemination through the development of a relational model opens up new avenues for research. However, the reliability and validity of this model, and the exact relationship between the variables, has not been tested using quantitative research methods. In addition, owing to research time and sampling limitations, research was conducted with Chinese audiences and materials only, and the research lacked a long-term follow-up investigation. The dynamically changing digital communication of handicraft ICH was also not considered. Therefore, future research should test the reliability and validity of large samples and the precise relationships between variables. Meanwhile, a long-term follow-up investigation of representative handicraft ICH digital communication is also necessary to obtain results that will verify the theory and be of practical significance.

## Supplementary Information

**Additional file 1: Appendix A**. Initial Interview Outline. **Appendix B**. CSSCI papers searched on CNKI (2016-2021). **Appendix c**. Open coding categories and original records.

## Data Availability

Not applicable.
