# Supplementary material for: Grounded theory-based model of the influence of digital communication on handicraft intangible cultural heritage
Source: Herit Sci. 2022 Aug 8;10(1):126. doi: 10.1186/s40494-022-00760-z (PMC9358110; doi:10.1186/s40494-022-00760-z)
Supplement: Supplementary file 1 — Additional file 1: Appendix A. Initial Interview Outline. Appendix B. CSSCI papers searched on CNKI (2016-2021). Appendix c. Open coding categories and original records. [file 40494_2022_760_MOESM1_ESM.docx]

***Appendix A***

**Initial Interview Outline**

1. Do you think all handicraft intangible cultural heritage communicators have an awareness of using digital media? If so, whom do you think does this?
2. What do you think about the current awareness of handicraft intangible cultural heritage? How do you view the problem of digital means and content cognition?
3. What are the obstacles regarding technical barriers and cultural understanding in the digital communication of handicraft intangible cultural heritage? Which methods could help reduce technical barriers and cultural conflicts?
4. Do you think the digital communication of handicraft intangible cultural heritage organically integrates technology and content?
5. Does the style of digital communication affect your impression of handicraft intangible cultural heritage? Does your original opinion change? Is your understanding of skill or culture deepened?
6. What problems should we pay attention to in fully exploiting the advantages of “digital”? Furthermore, how do we avoid the disadvantages?

***Appendix B***

**CSSCI papers searched on CNKI (2016-2021) (Keywords: intangible cultural heritage (ICH) & digital communication” or “traditional handicraft & digital communication” )**

| S/N | Literature | Authors | Publication | Publish time |
| --- | --- | --- | --- | --- |
| 1. P01 | 1. Reflection on the digital communication of intangible cultural heritage 2. 非物质文化遗产数字化传播的反思 | 1. Xie MW, Hou XF   解梦伟,侯小锋 | 1. Ethnic Art Studies 2. 民族艺术研究 | 2021 |
| 1. P02 | 1. The dissemination and development of intangible cultural heritage in the new media environment -- Comment on “Cultural Anthropology and intangible cultural heritage” 2. 新媒体环境下非物质文化遗产的传播与发展——评《文化人类学与非物质文化遗产》 | 1. Dong R 2. 董睿 | 1. Journalism Lover 2. 新闻爱好者 |  |
| 1. P03 | 1. Research on the digital dissemination of intangible cultural heritage in Yunnan Minority Areas 2. 云南少数民族地区非物质文化遗产的数字化传播研究 | 1. Zong W,Xiao Y, 2. Li GC 3. 宗蔚,肖洋,李刚存 | 1. Ethnic Art Studies 2. 民族艺术研究 |  |
| 1. P04 | 1. Research on image recording and effective utilization of intangible cultural heritage from the perspective of digital communication 2. 数字化传播视域下非物质文化遗产影像记录与有效活用研究 | 1. Nie HT,Han XY 2. 聂洪涛 韩欣悦 | 1. Social Sciences in Guangxi 2. 广西社会科学 |  |
| 1. P05 | 1. Dissemination of intangible cultural heritage of traditional handicrafts enabled by artificial intelligence 2. 人工智能赋能下的传统手工艺非物质文化遗产传播 | 1. Fan LG, Sun ZP 2. 樊传果,孙梓萍 | 1. Media Observer 2. 传媒观察 |  |
| 1. P06 | 1. New challenges and Countermeasures of digital communication of China's intangible cultural heritage 2. 中国非物质文化遗产数字传播的新挑战和新对策 | 1. Xue K, Long JY 2. 薛可,龙靖宜 | 1. Cultural Heritage 2. 文化遗产 | 2020 |
| 1. P07 | 1. The advanced direction of digital communication of intangible cultural heritage in the context of artificial intelligence 2. 人工智能背景下非物质文化遗产数字化传播的进阶路向 | 1. Jia J 2. 贾菁 | 1. Contemporary Communication 2. 当代传播 |  |
| 1. P08 | 1. The promotion path and mechanism innovation of cultural consumption -- a follow-up study of a typical case 2. 文化消费的提升路径和机制创新---一项典型案例的跟踪研究 | 1. Guan ZL 2. 管志利 | 1. Social Scientist 2. 社会科学家 | 2019 |
| 1. P09 | 1. Digital communication of traditional handicrafts in the new media era 2. 新媒体时代传统手工艺的数字化传播 | 1. Li D, Yu YZ, Zhang LJ 2. 李丹,余运正,张丽军 | 1. View on Publishing 2. 出版广角 |  |
| 1. P10 | 1. Thoughts on the Construction of Digital Communication Platform of Intangible Cultural Heritage in Qinghai Province 2. 青海省非物质文化遗产数字化传播平台构建思考 | 1. Ai F,Li JX,Zhang GX 2. 艾雰,李继晓,张国霞 | 1. Library Theory and Practice 2. 图书馆理论与实践 |  |
| 1. P11 | 1. Research on the digital communication strategy of intangible cultural heritage of ethnic minorities in Heilongjiang Province - from the perspective of "Internet +" 2. 黑龙江省少数民族非物质文化遗产的数字化传播策略探究——以“互联网+”为视角 | 1. Wang XM,Li H 2. 王晓敏,李晗 | 1. Heilongjiang National Series 2. 黑龙江民族丛刊 |  |
| 1. P12 | 1. Industry and number: Research on productive protection of intangible cultural heritage of ethnic minorities in Southern Guizhou 2. 产业与数字:黔南少数民族非物质文化遗产生产性保护研究 | 1. Li YL,Zeng YC 2. 李远龙,曾钰诚 | 1. Journal of South-Central Minzu University 2. 中南民族大学学报 | 2017 |
| 1. P13 | 1. Digitalized Protection of Shigong Dance as Intangible Cultural Heritage 2. 壮族师公舞非物质文化遗产数字化保护机制初探 | 1. Ouyang AH 2. 欧阳爱辉 | 1. Journal of Beijing Dance Academy 2. 北京舞蹈学院学报 |  |
| 1. P14 | 1. Study on the Adoption of Information Technology in Digital Transmission of Intangible Cultural Heritage 2. 非遗数字传播中的信息技术采纳研究 | 1. XU X, Sun YW 2. 许鑫,孙亚薇 | 1. Library & Information 2. 图书与情报 |  |
| P15 | 1. User Experience of Digital Transmission of Intangible Cultural Heritage 2. 徽州非物质文化遗产数字传播的用户体验研究 | 1. Guo HJ, Zhuang DH 2. 郭会娟,庄德红 | 1. Hundred Schools in Arts 2. 艺术百家 |  |
| 1. P16 | 1. Bibliometric analysis of intangible cultural heritage research in China from the perspective of communication -- a study of visual atlas based on CiteSpace 2. 传播学视域下我国非物质文化遗产研究的文献计量分析--基于CiteSpace的可视化图谱研究 | 1. Quan X 2. 权玺 | 1. Cultural Heritage 2. 文化遗产 | 2016 |

***Appendix c***

**Open coding categories and original records**

| **S/N** | **Category** | **most frequent original records（three each）** |
| --- | --- | --- |
| 01 | Digital environment cognition | 1. 1) 数字化传播的渠道是必不可少的，要擅加利用 2. Channels for digital communication are essential and must be exploited. 3. 2) 如果用这种数字技术的话，可能我们了解的更多，更全面一点 4. If we use this digital technology, we may know more and more comprehensively. 5. 3) 从内容到技术的表现，不是简单的介绍，是非常不容易的事 6. From content to technical performance, it is not a simple introduction. It’s so difficult. |
| 02 | Cultural adaptive cognition | 1) 任何一种文化都必须是要与时俱进，停留在原来的位置会有局限性  Any culture must keep pace with the times, because staying in the original position will have many limitations.  2) 现在最多的还是用那种娱乐的短视频形式来去展现  Now the most is to show it in the form of entertainment short video.  3) 感觉技术干技术的活儿，文化自顾自讲述，观众看了乏味也没有代入感  I feel that technicians do technical work while cultural scholars only talk about theories, and audiences feel bored after watching the video without any sense of empathy. |
| 03 | Communicator consciousness | 1) 他们还在以传统的方式思考这些问题，为什么不能改变下呢？  These craftsmen and communicators are still thinking about these issues in a traditional way, so why can’t they change the way of communication?  2) 手工艺人的传播意识是比较缺乏的，但媒体有这样的策划意识  The communication consciousness of craftsmen is relatively lacking, but the media has such planning consciousness.  3) 人人都有手机，随时可以发现各种美，都可以记录和上传  Everyone has a mobile phone, so you can find all kinds of beauty at any time, then record and upload. |
| 04 | Perspective of communication | 1) 存在感与数字体验发生冲突时，我们要充分理解和包容  When the sense of the presence of traditional handicrafts and the virtual sense of digital experience conflict, we need to fully understand and tolerate.  2) 文化就是让更多的人去知道、体验、传播  To understand culture is to let more people know, experience and spread.  3) 不管用什么技术，不要丢掉非遗本身的灵魂，不要把文化的根本丢掉  No matter what technology you use, don't lose the soul of intangible cultural heritage itself, and don't lose the root of culture. |
| 05 | Subject of communication | 1) 特别是当地的政府，现在都是尽最大限度最大能力去宣传  In particular, the local government is now doing its best to publicize.  2) 像博物馆这些大的这些机构在开始着手数字传播，比如说故宫  Large institutions like museums are starting to do digital dissemination, such as the Forbidden City.  3) 专业的人干专业的事情  Let professional people do professional things. |
| 06 | Content of the communication | 1) 比如做一些政府的这种创意周、文化周啊等等  For example, do some government creative weeks, cultural weeks, etc..  2) 中国水墨是非常传统的一种方式，利用3d 数字化技术把它实现出来  Chinese ink painting is a very traditional way, using 3d digital technology to achieve.  3) 中国的工笔其实是很静态的，但也可以把它从静态转化动态  Chinese fine brushwork is actually very static, but it can also be realized from static to dynamic. |
| 07 | Means of communication | 1) 在江苏苏州开了一个非遗文化展，宣传中很好利用了数字传播手段  An intangible cultural heritage exhibition was held in Suzhou of China, and digital communication methods were well used in publicity.  2) 清明上河图都开始着手去利用这种数字化的方式进行传播  Qingming Shanghe Tu also began to use digital means to spread.  3) 因为所有的东西都可以被泛娱乐化或短视频化，大家才会主动做  Because all things can be pan entertainment or short video, we will take the initiative to do it. |
| 08 | Effect of communication | 1) 旅游发现博物馆把数字化方式结合很好  The tourism Discovery Museum combines digital methods very well.  2) 音频方面喜马拉雅做的非常好，打开app输入非遗传播会发现很多内容  In terms of audio, Himalaya does a very good job. If you open the app to input intangible cultural heritage communication, you will find a lot of content.  3) 产业化和本土化结合，融入就会减少单独依靠技术进行传播的障碍  The combination of industrialization and localization will reduce the obstacles of relying solely on technology for communication. |
| 09 | Platform responsibilities | 1) 平台审核是第一道防线  Platform audit is the first line of Defense.  2) 平台在安全审核以及内容审核上，把关应该更严格  The platform should be more strict in security audit and content audit.  1) 各个平台有义务删除筛选内容  Each platform is obliged to delete the filtered content. |
| 10 | Technological rationality | 1) 以三维立体的形式来展现城墙，房间里360度环绕来观看  The city wall is displayed in three-dimensional form, and the room is surrounded by 360 degrees.  2) 充分利用技术的优势，借助它，而不是破坏艺术本身  Make full use of the advantages of technology and use it instead of destroying art itself.  3) 可能要需要一定的人去帮助进行传播，或者借助一定的平台去实现  It may need some people to help spread it, or with the help of a certain platform. |
| 11 | Crisis awareness | 1) 说技术至上是不对的，技术是手段，偏重技术而忽略内容是不对的  It is wrong to say that technology is supreme. Technology is a means. It is wrong to focus on technology and ignore content.  2) 如果不专业的人，随便上传自己的视频，会误导消费者  If unprofessional people casually upload their own videos, they will mislead consumers.  3) 技术上专业非专业的问题，这其实并不是最大的障碍  Technically, professional and non professional problems are not the biggest obstacle. |
| 12 | Value cognition | 1) 通过这种新的传播途径去了解的话，我觉得是加深了对文化意义的认知  Through this new way of communication to understand, I think it is to deepen the understanding of cultural significance.  2) 因为只有提高人的参与率，他才有继续传承的这种动力源泉  Because only by increasing people's participation rate can we have the power source to continue to inherit.  3) 不光中国人喜欢我们自己，外国人也很喜欢我们的李子柒和中国文化  Not only Chinese people like ourselves, but also foreigners like our Li Ziqi and Chinese culture. |
| 13 | Benefit connection | 1) 数字化慢慢普及之后，会慢慢习惯这种存在，习惯于这种便利性  After the gradual popularization of digitization, we will get used to this existence and convenience.  2) 数字化传播的形式有些不太符合年轻人的观点，有一定的限制  Some forms of digital communication are not in line with the views of young people, and there are certain restrictions.  3) 唐宫夜宴技术确实有达到，所以大家觉得非常好看非常美，愿意去传播  Tang Palace Banquet technology is really powerful, so everyone feels very beautiful and is willing to spread it. |
| 14 | Emotional reaction | 1) 我觉得数字传播更有亲和力，距离近了  I think digital communication is more friendly and closer.  2) 有些非遗视频我仅仅是看看，仅停留在点赞转发，很少评论  After watching videos and animations of handicraft intangible cultural heritage, I just liked and forwarded it, but rarely commented.  3) 现有的趣味性、体验性、简便性稍差一些，观众获取不到那么多的感受  The existing interest, experience and simplicity are slightly worse, and the audience can't get so many feelings. |
| 15 | Institutional aspects | 1) 权威专家应该有更高的曝光  Authoritative experts should have higher exposure.  2) 我们初中每周都去工艺基地练习， 一旦有新的数字体验项目，我们会很高兴，希望下次再去  In junior high school, we went to the craft base to practice every week. Once there was any new digital experience project, we would be very happy and hoped to go again next time.  3) 国家是倡导去做一些国风类或者跟传统文化息息相关的内容  The state should advocate to do some national style or content related to traditional culture. |
| 16 | Social aspects | 1) 泛娱乐化时代里面，我觉得还是需要提高全民的艺术修养  In the era of Pan entertainment, I think it is still necessary to improve the artistic cultivation of the whole people.  2) 学校和家庭教育上，要善于发掘人才，培养有能力有专长的传承者  In school and family education, we should be good at exploring talents and cultivating inheritors with ability and expertise.  3) 需要一个比较好的平台和机构，有这样一个资金去做这个事  We need a better platform and organization with such funds to do this. |
| 17 | Opportunity | 1) 李子柒的手工艺视频记录，数字内容和新媒体选择不能取悦所有人，但无论如何，前提是要有正确的沟通观念。  Just like Li Ziqi’s live broadcast of production videos of various crafts, digital content production methods and new media choices cannot please everyone. But in any case, the premise is to have a correct communication concept.  2) 这个时候去做非遗、国风其实是一个特别顺应潮流的事情  At this time, to do intangible cultural heritage and national style is actually a special thing that conforms to the trend.  3) 你降低门槛的同时，其实是让更多的量进来  When you lower the threshold, you actually let more quantity in. |

*Note: Data collection was based on Chinese audiences (including overseas students), so the original in-depth interview records were in Chinese.*
